# Study of Machine Learning for Cloud Assisted IoT Security as a Service

**DOI:** 10.3390/s21041034

**Published:** 2021-02-03

**Authors:** Maram Alsharif, Danda B. Rawat

**Affiliations:** Data Science and Cybersecurity Center, Howard University, Washington, DC 20059, USA

**Keywords:** machine learning, cloud assisted IoT security as a service

## Abstract

Machine learning (ML) has been emerging as a viable solution for intrusion detection systems (IDS) to secure IoT devices against different types of attacks. ML based IDS (ML-IDS) normally detect network traffic anomalies caused by known attacks as well as newly introduced attacks. Recent research focuses on the functionality metrics of ML techniques, depicting their prediction effectiveness, but overlooked their operational requirements. ML techniques are resource-demanding that require careful adaptation to fit the limited computing resources of a large sector of their operational platform, namely, embedded systems. In this paper, we propose cloud-based service architecture for managing ML models that best fit different IoT device operational configurations for security. An IoT device may benefit from such a service by offloading to the cloud heavy-weight activities such as feature selection, model building, training, and validation, thus reducing its IDS maintenance workload at the IoT device and get the security model back from the cloud as a service.

## 1. Introduction

The revolution of computer and network technologies pushed the Internet to take a great leap towards unprecedented services linking different scales of smart devices with individuals and entities in what we call the Internet of Things (IoT). The IoT ecosystem embraces millions of smart devices that are heterogeneous in design, role, and ownership. Smart devices can take different forms such as sensors and light switches in smart homes, a public webcam, a vehicle, or traffic lights as a part of intelligent transport system [1].

The core of IoT devices is built around embedded systems which combine computer hardware and software to play specific role or to perform specific function as part of a larger system. A system may be operating in different environments such as, but limited to, personal, industrial, educational, and transport [2].

The openness to the public exposes those devices to different security risks that exploit their vulnerabilities and consequently affect their integrity, confidentiality, and availability [3]. Even with the continuous expansion of IoT, attacks become more prevalent and complex. So, the protection of devices in such a heterogeneous environment became a great concern and a challenge more than ever before [4]. However, due to devices’ limited computing resources, traditional security mechanisms become heavy-weight for IoT devices to function efficiently [5]. Accordingly, the devices are used without sufficient protection making them prone to direct attacks or vulnerable to compromise by attackers to launch attacks on third parties (known as botnets) [6]. In one incident, multiple IoT devices were used to start a distributed Denial-of-Service (DDoS) attack on an American Internet services company that made it impossible for many customers to access certain Internet services [5].

Intrusion detection systems (IDS) have gained the interest of researchers to secure IoT devices against launched attacks from adversaries. Researchers adopted machine learning (ML) techniques to detect network traffic anomalies caused by known as well as newly introduced attacks, and to warn the appropriate network control nodes to block such traffic [7]. Proposed techniques normally focused on the functionality metrics depicting their prediction effectiveness but overlooked their operational requirements.

Machine learning (ML) is characterized by its hunger for computing resources throughout all its phases. For intrusion detection systems, extracting features from a connection’s packets is a fundamental activity for building, testing, and running the system. Collected data samples require cleaning and scaling. Building a model requires attribute categorization, fitting, and validation. All those activities should be carried out in timely sequence otherwise slipping risky packets undetected is inevitable [8]. On the other hand, integrating ML within embedded systems operation should consider the diversity of their computing resources such as the CPU architecture, the provision of graphical processing unit (GPU), the size of the physical memory, and the network connectivity [2]. Those factors undoubtedly affect the operational feasibility of ML-IDS on IoT devices in terms of prediction throughput, packet miss rate, and computing resources utilization.

The research community has been focusing on improving the accuracy of ML models in the detection of traffic anomalies and specific attack types and gave little consideration for their operational feasibility. To bridge the gap between the functional and the operational feasibility of ML-IDS, we propose a framework of managing ML based IDS that supports the adaptation of ML models to the heterogeneous operational environments that exist in the IoT. We attempt to reach a controlled compromise between ML metrics and operational metrics. We adopt cloud support to our framework to provide the appropriate operational scale supporting the huge IoT devices population.

The framework is composed of two interacting layers: a cloud layer and IoT device layer. A cloud-based service, Cloud Model as a Service (MaaS), is integrated in the cloud layer to carry out heavy-weight tasks on behalf of the IoT devices, including feature selection, model building, training, validation, in addition to model distribution to IT devices. The service would potentially adapt the developed models, to best fit each device’s operational resources, by optimizing the training features fitted in the device’s model. In the IoT device layer, the devices are limited to lighter weight activities such as attribute extraction from network traffic and IDS model prediction of anomalies. The framework groups IoT devices of identical roles and resources into manageable sets to optimize the cloud service workload. Anomalies detected by the devices are propagated to the cloud layer for integration in the training dataset, and for building samples’ batches for distribution to devices capable of performing incremental training.

The proposed framework reliefs resource-limited IoT devices from excessive ML activities, contributing to ML-IDS application feasibility. It also guarantees seamless distribution of updated models and samples’ batches to IoT devices, thus providing continuous protection of IoT devices against emerging new attacks.

In Section 2 we present relevant research work on resource-limited IoT devices and cloud adaptation to secure IoT devices. Section 3 we introduce our proposed Cloud MaaS concept for IoT security. Then we present in Section 4 and Section 5 the operational considerations of the cloud framework, and the results of our ongoing research. We conclude this paper with remarks and proposals in Section 6.

## 2. Related Work

Anomaly Intrusion detection methods require reliably tested and verified datasets to guarantee accurate performance [9]. There are 11 criteria that have been identified in research to characterize the quality of the dataset: complete network configuration; complete traffic; labeled dataset; complete interaction; complete capture; available protocols; attack diversity; anonymity; heterogeneity; feature set; and metadata [10]. In order to optimize and adjust the dataset to be ready for processing by machine learning algorithms, a set of considerations was proposed in [9] that contribute to a high detection rate of the IDS for all attacks referenced in the data set:

- Null values are not accepted by machine learning algorithms.
- The type of features values in the dataset that best serves ML algorithms.
- The correlations between features in the dataset. High correlations would mean redundant features.
- Identifying the important features values for classifying specific attacks. Some features may not influence the detection of analyzed attacks.
- How balanced the dataset and identifying the most effective method to balance the dataset. Unbalanced dataset cause ML algorithm to be biased to a larger set of instances for a specific attack.
- Identifying the relevant normalization function that produces the best detection rate, since not all normalization functions suit all datasets.

The creation of a training set may face difficulties. The protection of web applications, for example, should contain all combinations of endpoints devices and cookies due to their high number [11]. Overcoming such difficulties is crucial for the dataset quality to perform high accuracy classification.

Datasets may be classified according to network traffic granularity upon which attributes are extracted, namely, packet-based and flow-based [12]. UNSW-NB15 dataset supports packet-based detection [12], while CICDDoS2019 supports flow-based training and detection [13]. CICIDS 2017 [14], ISCX 2012 [15], and TUIDS [16] are labeled datasets supporting both traffic granularities.

IoT devices have vulnerability to many of the aforementioned attacks which is related to either: limitations in their computational resources, lack of effective encryption, insecure web services, lack of effective authentication and authorization mechanisms, and heterogeneity. This makes applying security mechanisms uniformly in IoT devices a challenging issue [17]. In the following we review relevant research efforts aiming to leverage IoT security. We highlight key features of each citation regarding performance metrics for both IDS functionality and the underlying operational platform [18]. Verma et al. conducted performance assessment of seven machine learning classification algorithms (RF, daboost, gradient boosted machine GBM, extremely randomized trees ERT, classification and regression trees, and MLP) [7]. The performance metrics were accuracy, specificity, sensitivity, false positive rate, and area under the receiver operating characteristic curve. The presented measurements were limited to the average response time taken by different classifiers for classifying a single instance.

Bakhsh et al. proposed adaptive intrusion detection and prevention system for the IoT devices utilizing agent technology to support portability, rigidness, and self-started attributes [3]. The system was meant to sit in the middle of the network layout, serving its IoT devices. This was a hybrid solution that combines both the detection of misuse and anomaly and integrates both host-based and network-based functionality. The system analyzer applies pre-defined detection rules to captured packets, and blocks traffic when an attack or intrusion takes place. The research did not provide any performance measurements for either the IDS or its running platforms. Anthi et al. proposed a predictive and adaptive network-based IDS system tailored for IoT ecosystems, adopting both signature and anomaly-based detection [19]. The research experimented with simple versions of DoS attacks such as SYN and UDP Flood Attacks. The testbed consisted of small set of IoT devices with IDS monitoring the network and running on a Mac book. Such devices were connected to a Sky router forming realistic IoT ecosystem. The research was not clear on the model details and achieved moderate attack detection results. Susilo et al. examined machine learning Random Forests (RF), Support Vector Machine (SVM) and deep learning Multilayer Perception (MLP), Convolutional Neural Network (CNN) algorithms for network-based IDS utilizing the GPU of an advanced desktop computer [20]. The research aimed at improving processing speed by increasing the change in batch size when building the MLP. Speeding up the calculation process by 1.4–2.6 times was achieved, while the CNN achieved speeding up the calculation process 1.8–2.4 times.

Soe et al. studied the potential of Raspberry Pi 3 Model B for building and executing an IDS based on UNSW-NB15 dataset having nearly 175,000 instances with 49 features [21]. The research applied a correlation-based feature selection (CFS) to limit the number of selected features to only 7 for 9 attack types, thus enabling the device to train J48 decision tree algorithm using the complete dataset. The CFS achieved speed up of 100% for training and testing (80, 8 s respectively), but reduced ML metrics by 10% (0.8) when compared to non-CFS model. For Naive Bayes Classifier (NB) algorithm, the CFS achieved speed up of 300% for training and testing (10, 10 s respectively), but produced fluctuating and yet reduced ML metrics (0.1–0.8) for different attack types. Thamilarasu et al. discussed the practicality of deploying ML-based IDS for resource-limited IoT networks and proposed a Deep Learning Neural Network (DNN) model, composed of layered 34 nodes with 3 hidden layers and trained it for 150 epochs [1]. The model was implemented on Raspberry Pi 1 Model B, trained and tested using locally generated dataset of 6 packet-based features in 40,000 training instances and 20,000 test instances. The model achieved over 90% success rate for ML metrics. The research did not evaluate the prediction performance of the testbed under realistic network traffic, though demonstrated the effectiveness of the model in terms of anomaly detection for specific attack scenarios: blackhole attack, opportunistic service attack, DDoS attack, sinkhole, and wormhole attacks.

Doshi et al. examined five ML algorithms KNearestNeighbors (KNN), Lagrangian Support Vector Machine (LSVM), Decision Tree (DT), RF, DNN using Raspberry Pi 3 Model B, and integrating Scikit-learn Python library, and Keras (for DNN) [22]. The research considered 11 features of packet (stateless mode) and flow-based (stateful mode) instances from locally generated dataset and achieved 0.99% ML metrics for all algorithms except for the LSVM (less Recall score). They made assumptions of specific behaviors IoT network (e.g., limited number of edge node and fixed time intervals separating packets) when performing feature selection resulting in better accuracy DDoS detection in IoT network traffic. They claimed that stateful features save flow information for very short time, thus requiring least memory to support deployment on routers.

Cloud computing, in contrast to the traditional development of centralized network anomaly detection architectures, exploits the concept of Software as a Service (SaaS) for the benefit of anomaly detection in a rather distributed environment. SaaS enables providers to rent their security services to users to offload complex tasks to the cloud facilities over the Internet and reduces the cost of managing new user infrastructures.

Yassin et al. proposed cloud-based architecture for intrusion detection service using signature-based anomaly detection [23]. Such services receive captured traffic instances from client’s own cloud and analyzes them within the SaaS cloud, and reports the outcome back to the client. Chawla et al. proposed the Security as a Service (SaaS) concept in which anomaly detection service is provided using dedicated IoT devices placed in the center of user cloud to probe and detect intrusions targeting or generated from each individual user device within the cloud [24]. Keegan et al. reviewed the application of parallel processing paradigm (MapReduce) and network flow applications (NetFlow, sFlow, OpenFlow, and IPFIX) for conducting different ML activities in parallel using cloud processing clusters [17]. Though the cloud computing provided extensible resources for handling large datasets for training and classification, many drawbacks were reported regarding flow analysis tools, high packet drop rate, high false positive rate, and limited parallelism inherent in some ML models.

In our research, we introduce cloud computing and adaptive modeling with a different perspective to fill in the gaps of existing research regarding cloud and adaptive techniques. We introduce a framework integrating cloud services, that are globally accessible rather than being centric to local networks, and correlating IoT device operational characteristics to key ML activities involved in the construction of ML model thus generating adaptive models that achieve acceptable detection and operational feasibility metrics.

## 3. The Proposed Cloud MaaS Cloud Architecture

**Figure 1:**
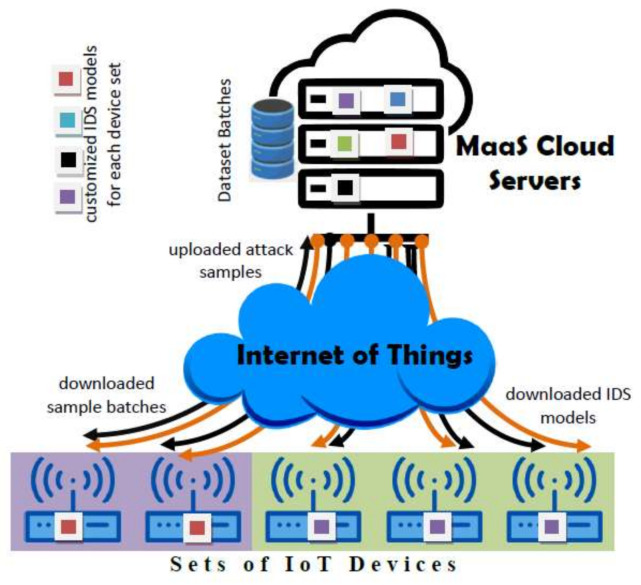
Cloud-based Adaptive for intrusion detection systems (IDS) Network Architecture.

The proposed architecture is composed of two main layers interacting throughout the IoT (see Figure 1): the cloud service layer responsible for the management of ML-based IDS models, and the devices layer organizing devices into manageable sets each running identical IDS model. In the following we elaborate on the architecture layers and their interactions.

### 3.1. Cloud Service Layer

It represents the cloud part in the Cloud MaaS which utilizes the existing cloud services SaaS (Software as a Service) to undertake the building and training of models targeting each set of devices. The service would also collect anomaly and attack instances from the participating devices, group them into batches, and distributes them to relevant sets. Due to the heterogonous design and role of the millions of edge devices hooked to the IoT, one can imagine the enormous workload such a service would bear, requiring the exploitation of the huge cloud computing resources.

**Figure 2:**
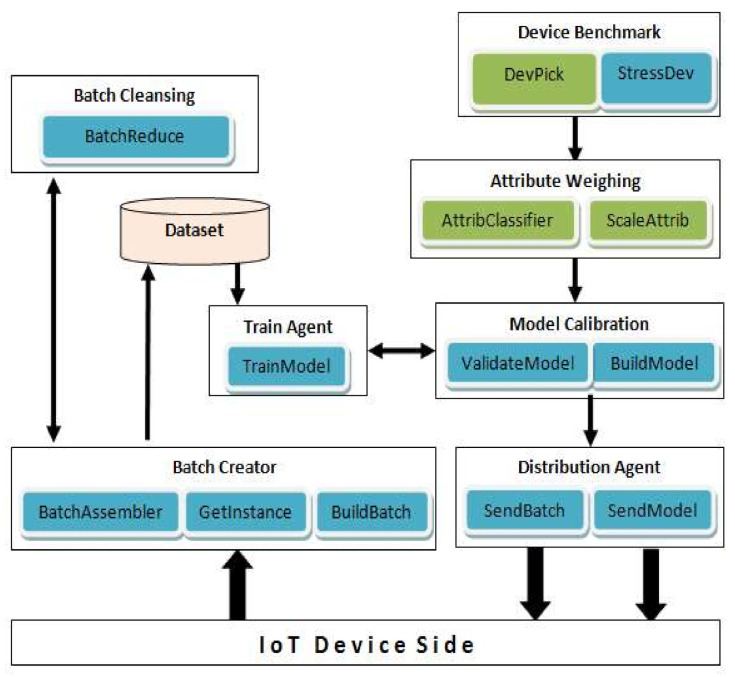
Cloud MaaS Functional Components.

The role of Cloud MaaS may be assigned to a single or multiple nodes in the cloud. This is dependent on the node workload pertaining to assigned device sets and the number of devices they contain. So multiple nodes may split device sets amongst themselves to balance their workload distribution. The functional components of the Cloud MaaS nodes are depicted in Figure 2:

Device Benchmark—specifies the available resources (e.g., CPU and memory) and the average network traffic for IDS model operation on the edge device. This component installs the benchmark agent on a selected device member of each device set and collects agents’ measurements for the Attribute Weighing component to perform.

Attribute Weighing—applies operational weight to each important feature involved in the model building (based on required FLOPS and storage size for each feature). Such weights indicate the feature extraction workload in edge devices and should influence model building. The weighted features are made available to Model Calibration component.

Model Calibration—updates the model parameters based on the operational weights of important features and may exclude important features that prove to overload relevant edge devices and cause packet inspection miss. Example: equal operational weights of 0.1 for 15 features would mean 50% overload thus selecting at most 10 features that provide maximum classification and detection accuracy for building the model.

Batch Creator—collects reported instances from devices and merges them into batches for incremental training. Batches have preset size to be fulfilled prior to being applied in Train Agent.

Batch Cleansing—tailors generated batches to each set device. This implies removing device’s reported instances from the corresponding set batch to avoid replication at device’s side.

Distribution Agent—When a new model is created for a particular device set, this component will distribute the model to the set’s participating devices.

Train Agent—trains new models with relevant compiled and updated dataset, otherwise it incrementally trains existing model with new batches.

### 3.2. IoT Device Layer

This layer groups edge devices into sets where each set is characterized by identical devices role and operational platform (identical HW and SW configurations). This grouping regulates the involvement of the cloud service which we shall discuss herein. An edge device would download its updated model parameters from the cloud service, use it for predicting anomalies and attacks, and inform the cloud service of detected anomalies or attacks instances. The device may use the same instances for local incremental training.

**Figure 3:**
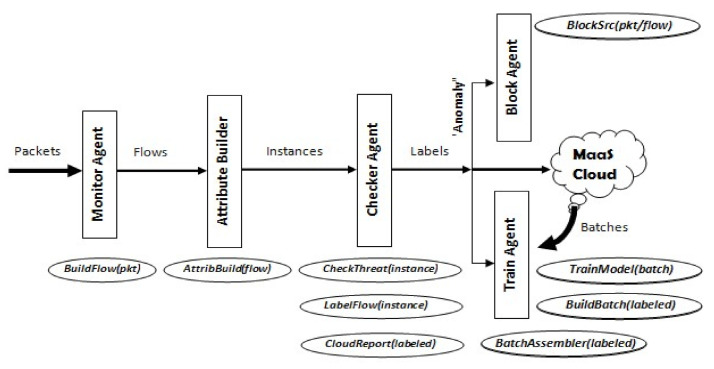
Proposed IDS Structure for IoT Devices.

The IDS running on IoT edge devices is composed of 5 main functional components (see Figure 3). The IDS may run in two distinct modes: packet-based or flow-based. The first implies analyzing network traffic on packet basis upon which model prediction is applied to detect if a packet presents any anomaly. The latter implies aggregating unidirectional packets forming a session or a connection with a remote Internet node, into a flow set presenting a unit for analysis. Either mode of operation requires a training dataset supporting either mode. The functional components are almost identical in both modes of operation. Here we elaborate on the role of each component:

Monitor Agent—is responsible for capturing incoming network packets, passing them to the first analysis component, the Attribute Builder, either in their individual packet form, or assembled in flow sets.

Attribute Builder—is responsible for extracting attribute data from either single packets or flow sets and generating what we call “instance data” which will be processed by the ML model.

Checker Agent—passes each instance data to the ML model for anomaly prediction. If anomaly is detected, an alarm is initiated on the device, and the source of the incoming packet/flow is blocked while the packet (when in packet mode) is dropped. The instance causing the alarm is then reported to the cloud node for distribution to other devices.

Train Agent—incrementally trains the model using locally detected instances labeled as “anomalous” but first assembles them into batches to be applied to the model when the device is idle. This agent also receives batches of labeled instances from the cloud node (excluding the device’s reported instances) to train the model as well.

Block Agent—takes care of blocking traffic received from anomaly sources. This may be performed on the device itself or reported to a firewall for taking appropriate action. Figure 3 shows the proposed IDS architecture and depicts the main functions associated with each component.

## 4. Research Experiments and Considerations

To validate the cloud-based architecture, several considerations are adopted in order to evaluate the architecture effectiveness, and to determine its limitations. For example, IDS operation modes (e.g., Packet and Flow-based) would influence the selection of the sample dataset. Continuous training of models in the cloud or the device layer influences the model selection (e.g., incremental vs. full dataset). Further, different alternatives for benchmarking real embedded systems are considered for setting up virtual testbeds and for building the relevant cloud layer. In the following we elaborate on those considerations.

*Packet-Based IDS Operation*: This mode is stateless and requires the least memory for its operation. We endorse this mode for IoT devices with limited memory to buffer session packets for inspection. This mode will persist during training or prediction phases.

*Flow-Based IDS Operation*: This mode is stateful, requiring more memory for grouping and analyzing unidirectional packets belonging to the same flow session. In this mode, packets are not dropped but rather the flow source is blocked upon anomaly detection. When combined with packet mode in a single pipeline, we create a hybrid mode for deeper inspection of traffic and enhance detection efficiency.

*Selection of ML Model*: The incremental model learning requirement on edge IoT devices necessitates the adoption of a supporting ML models. So, we consider evaluating models such as: SVM, MLP, RF, CNN, DNN, and Ensemble methods.

*Training Datasets*: We are building our models based on public datasets that support flow-based labeled attributes such as CICDDoS2019 dataset, or both packet and flow-based labeled attributes, such as CICIDS 2017, ISCX 2012, and TUIDS labeled datasets [16,25].

*Research Environment Setup*: Conducting this research requires a multifaceted setup, partly done in real computing environment (Raspberry Pi and Coral) embedded systems. The research is also conducted in virtual environment on user computing facilities with virtualization tools (Oracle’s VirtualBox) and collaboration cloud services (Google’s Colab).

*Testbed Benchmarks*: We are considering available benchmarks on Raspberry Pi, including Linpack, TensorFlow when building the virtual environment to correctly mimic the actual operational environment.

*Benchmarking IoT Devices*: The benchmarking agent of edge devices is built using “psutil” library in Python code. Different applications depicting realistic roles of such devices are being simulated using stress tools “stress” and “stress-ng”. The simulation provides accurate calibration of available device’s computing resources to achieve realistic measurements of ML models performance.

## 5. Performance Evaluation and Discussion

This section presents the investigation results that highlight heavy duty activities such as model fitting with large feature set using moderate-size dataset. Different ML models are considered concluding the need to offload such activities to the cloud layer. The investigation estimates the sizes of updated models to be downloaded frequently to IoT devices from the cloud layer, and the results depict their feasibility. Reducing the number of fitted features on the other hand proved the concept of tailoring models to suit resource-limited IoT devices.

The investigation testbed is a VirtualBox guest simulating Raspberry Pi 4 on a Windows host computer equipped with Intel Core-i7 @ 2.4 GHz and 8 GBytes of RAM. The virtual guest is set to a single CPU with execution cap set to 20% to match realistic benchmarks mentioned in this paper.

The simulation of the quad core CPU architecture of Raspberry Pi 4 was achieved by enabling the VirtualBox acceleration feature, utilizing 4 host CPU cores. With 4 GB of memory, we were able to process 725,165 instances with 88 features labeled for benign and 3 attack samples: Syn, UDP, UDPLag in the CICDDoS2019 dataset. The investigation was conducted using Python notebooks run on the virtual machine excluding GPU acceleration available on Raspberry Pi due to infeasibility of simulating it on the virtual environment.

We applied the RandomForest Classifier (RFC) for feature selection and we were able to identify 15 important features for our investigation. RFC run consumed ~60% of memory utilization and ~95% of CPU utilization when processing 70% of the training data, in 3846 s. This demonstrates the operational infeasibility of running such classifier for feature classification even on a resourceful device such as Raspberry Pi 4.

In this investigation, we considered 5 ML models referenced recently by many researchers (e.g., NB, DT, KNN, Logistic Regression (LR), and DNN). The investigation was centric around the operational characteristics: the CPU processing time for training different models, no involvement of a GPU, and the model size in memory. It also included ML metrics evaluation for each model: Cross Validation Mean Score; Model Accuracy; F1-Score; Classification Precision; and Recall.

Table 1 depicts the investigation results of operational characteristics of the 5 models. Model training shows only the consumed CPU time based on the 15 classified features and 4 preselected features run on both the virtual and the host machines.

Applying pre-selected 4 key features when fitting the models rather than relying on the RandomForestClassifier for selecting the 15 important features, achieved tens of folds of speed up in terms of CPU time (i.e., operational improvement) of all models except for the KNN’s degraded performance, and the DNN’s 50% improvement. An example of processing time improvement is demonstrated by the NB model requiring 1.41 s of CPU time for training based on the 4 features compared to 124 s when processing the 15 features. The NB achieved the best performance, followed by DT, DNN, LR, and KNN (the worst). The DNN was configured in two cases with 15 and 4 input layers respectively with 1 hidden layer. In order to avoid overfitting, the former executed 6 epochs while the later executed 4, for 40 ms each. The obtained results for the five models using the pre-selected four key features showed minor degradation of some ML metrics compared to Table 1 results, though model accuracy was almost fixed.

The Naive Bayes Classifier outperforms the rest requiring the least CPU time, followed by Decision Tree algorithm and Logistic Regression (the slowest). From Table 1, we deduce that KNN is impractical to use in IoT devices, from the operational perspective, since the model’s size is proportional to the dataset size, and it is sensitive to noise in the training data. The LR model had the smallest size, followed by DT, NB, and then DNN. Downloading updated models of few Kbytes in size (except for the KNN) from the cloud MaaS prove to be practical as it requires few seconds to complete using current network technologies.

Table 1 also depicts the potential of building the models in the cloud layer, represented by the testbed computer, where tens folds of speed up are achieved for some models.

As for ML metrics, Table 2 depicts the 5 ML metrics measurements for the 5 model types, trained with the 15 classified features and the 4 preselected features, indicating their close performance in terms of Cross Validation Mean Score and Model Accuracy. The F1-Score, Classification Precision, and Recall rows indicate each model’s performance for predicting the labeled feature, with four consecutive numbers depicting benign; and Syn, UDP; UDPLag attack classes respectively (i.e., F1-Score for NB model is 0.95 for benign, 1.0 for Syn attack, 1.0 for UDP attack, and 0.62 for UDPLag attack when considering the 15 classified features).

Classification Precision is the ratio of correctly predicted positive observations to the total predicted positive observations, so a value of 1.0 means no false positive predictions, while 0.0 value means model failure to identify relevant labeled feature samples. Recall is the ratio of correctly predicted positive observations to all observations in actual class, so a value of 1.0 means no false negative predictions, while 0.0 value means model failure to identify relevant labeled feature samples. F1 Score is the weighted average of Precision and Recall, so a value of 0.0 requires reference to both Classification Precision and Recall values of the relevant labeled feature to identify model weakness. Model Accuracy is the ratio of correctly predicted observation to the total observations and the closer value to 1.0 means higher model accuracy in its predictions. Fewer samples per labeled feature minimizes its influence in the overall Model Accuracy, so F1-Score needs to be evaluated alongside to assure uniform model performance for all labeled features. Having around 1% samples for UDPLag attack in the training samples demonstrates this fact for the NB, KNN and LR models.

The LR model demonstrates acceptable Model Accuracy while failing to predict UDPLag attacks (e.g., having 0.0. for that attack label in both Classification Precision and Recall).

The NB, DT, KNN, and LR models vary in their results for the 4 labeled features. The DT outperforms the rest with perfection throughout the 4 categories, while LR provided the worst performance. The introduction of the 4 preselected features caused degradation of the performance of the NB model in identifying benign and UDPLag samples, while resulted in negligible changes in other models’ performance.

Neural Network model achieved reasonable functional and operational performance and outperforms the rest in continual training activity since it is the sole model of the five that supports incremental training.

This drives the conclusion that optimizing the number of features without sacrificing detection performance, and consequently reducing the storage size of the training dataset supports the concept of downloading sample batches from the cloud layer to resource-limited IoT devices whenever local device training is required. Such optimization also reduces the workload of feature extraction from captured traffic samples for attack prediction.

Our future work includes the implementation of other machine learning techniques [17,22,25] including reinforcement learning in IoT [26] and implementation of resilience machine learning for Cyber Physical Systems and IoT [27].

## 6. Conclusions and Future Work

This paper presented a cloud assisted ML-based security solution for resource-limited IoT devices that achieves the implementation of efficient ML-based IDS for their protection. Cloud MaaS framework is proposed to offload heavy weight ML activities to dedicated cloud computing resources and to organize IoT devices into manageable matching sets. Devices in a set receive updated IDS model from the cloud optimized for their available computing resources and their assigned role workload. IDS operation would then be limited to feature extraction from network traffic and anomaly prediction. Detected anomalies are reported to the cloud service for further examination and continual model training. The virtual testbed results of different ML techniques based on two sets of features proved the effectiveness of reduced feature set in terms of both model detection efficiency and operational feasibility. This proves the concepts of MaaS for managing models that match computing resources of a diversity of IoT devices. We are working on further verification of the Cloud MaaS concept on realistic embedded systems and validating the cloud service algorithm for different sets of IoT devices.

## Figures and Tables

**Table 1:** Operational Metrics of Machine learning (ML) Models.

	NB	DT	KNN	LR	DNN
Model Training (CPU Time in Sec.) on Virtual Device					
(15 features)	124	204	441	1395	240
(4 features)	2	6	2500	204	160
Model Training (CPU Time in Sec.) on Host Computer					
(15 features)	5	8.9	25	75	15.6
(4 features)	0.17	0.34	104	11	9.2
Model Size (Kbytes)					
	3.5	3.2	372000	2.3	18

**Table 2:** ML Metrics of ML Models.

	NB	DT	KNN	LR	DNN
Model Accuracy	0.998	0.999	0.999	0.992	0.826
Mean Squared Error	0.998	0.999	0.999	0.993	N/A
F1-Score					
(15 features)	0.95/1.0/1.0/0.62	1.0/1.0/1.0/1.0	0.99/1.0/1.0/0.59	0.30/1.0/0.97/0.0	N/A
(4 features)	0.26/1.0/0.99/0.0	0.95/1.0/1.0/0.75	0.94/1.0/1.0/0.70	0.32/1.0/0.99/0.0	N/A
Classification Precision					
(15 features)	0.93/1.0/0.99/0.90	1.0/1.0/1.0/1.0	0.99/1.0/1.0/0.75	0.83/1.0/0.97/0.0	N/A
(4 features)	0.93/0.99/0.98/0.0	0.98/1.0/1.0/0.74	0.98/1.0/0.99/0.75	0.95/0.99/0.98/0.0	N/A
Recall					
(15 features)	0.97/1.0/1.0/0.48	1.0/1.0/1.0/1.0	0.98/1.0/1.0/0.48	0.18/1.0/1.0/0.0	N/A
(4 features)	0.15/1.0/1.0/0.0	0.92/1.0/1.0/0.76	0.90/1.0/1.0/0.66	0.19/1.0/1.0/0.0	N/A

## Data Availability

Not applicable.
